# DEVELOPMENT OF A COMMUNITY-ENGAGED DEMENTIA EDUCATION PROGRAM FOR THE VIETNAMESE AMERICAN COMMUNITY

**DOI:** 10.1093/geroni/igad104.0167

**Published:** 2023-12-21

**Authors:** Christina Miyawaki, Kim Nguyen, Angela McClellan, Tuong-Vi Ho

**Affiliations:** University of Houston, Houston, Texas, United States; University of Houston, Houston, Texas, United States; Baylor University, Waco, Texas, United States; Texas Woman’s University, Houston, Texas, United States

## Abstract

Despite the life-threatening escape from Vietnam, Vietnamese refugees’ health data are scarce. To fill this knowledge gap, we developed the Vietnamese Aging and Care Survey (VACS) and examined the health of community-dwelling older Vietnamese (≥65 years) in Houston, Texas, the 2nd largest Vietnamese-populated metropolitan area in the U.S. The data revealed their adverse health conditions including a high prevalence of cognitive impairment. We proposed a Community-Engaged Dementia Education Program to improve the community’s dementia literacy based on the Cultural Exchange Model as a conceptual framework. Using Edwards’ 9-stage Community Readiness Model, 14 Vietnamese key informants assessed their current literacy level (Aim 1). To develop dementia educational materials (Aim 2), the informants formed focus-group discussions. We used thematic analysis to analyze the community’s understanding of dementia and developed educational materials. Key informants were aged 58 years (mean), Vietnam-born (93%), married (79%), college-educated (72%), women (86%), and full-time employees (79%). They lived in the U.S. for 39 years and in Houston for 30 years. Themes such as the Vietnamese community’s low dementia literacy, their perception of dementia as a normal part of aging, and suggestions for introducing dementia conversations to the community emerged. We considered a one-pager as appropriate educational material, and included culturally- and linguistically-appropriate content and its translation, a target population’s image, and dementia warning signs encouraging them to see their doctors for cognitive check-ups. Our next steps include utilizing local ethnic social media, disseminating the one-pager, and offering a “Brain Booth” at various Vietnamese health fairs.

